# Revisiting the Elusive Hepatitis C Vaccine

**DOI:** 10.3390/vaccines9020114

**Published:** 2021-02-02

**Authors:** Stephen M. Todryk, Margaret F. Bassendine, Simon H. Bridge

**Affiliations:** 1 Faculty of Health & Life Sciences, Northumbria University, Newcastle upon Tyne NE1 8ST, UK; 2 Translational &Clinical Research Institute, The Medical School, Newcastle University, Newcastle upon Tyne NE2 4HH, UK

The impactful discovery and subsequent characterisation of hepatitis C virus (HCV), an RNA virus of the flavivirus family, led to the awarding of the 2020 Nobel Prize in Physiology or Medicine to Harvey J. Alter, Michael Houghton and Charles M. Rice [1]. However, despite the significant advances recognised by this Nobel prize, an effective HCV vaccine remains elusive. The recent success of vaccines against SARS-CoV-2, developed with unprecedented speed, has shone a bright light on the vaccination process for protection against viral threats and may provide renewed impetus for the development of vaccines for other viruses. HCV infection remains a major global health problem as approximately three quarters of those infected develop chronic infection, leading to morbidity and mortality due to progressive liver fibrosis, cirrhosis and cancer. The World Health Organization (WHO) estimates that 71.1 million people are living with chronic HCV, resulting in over 400,000 deaths every year. In 2016, the WHO adopted a global strategy with the aim of eliminating viral hepatitis as a public health problem, comprising targets to reduce new viral hepatitis infections by 90% and reduce deaths due to viral hepatitis by 65% by 2030. Despite the use of highly effective antiviral drug combinations (direct-acting antivirals (DAAs)) and the adoption of treatment as prevention in resource-rich healthcare settings, there are an estimated 2–4 million new HCV infections annually, and the consensus is that the WHO target is unachievable without other measures, including an effective vaccine. A safe and affordable vaccine would particularly provide a realistic proposition in low- to middle-income countries with resource-constrained healthcare settings, which bear the brunt of chronic infections (>80%). In addition, a vaccine would prevent infection or reinfection in high-risk groups in all countries. These groups comprise people who inject drugs, men who have sex with other men, prisoners, and people in receipt of poor medical and other procedures (i.e. unsafe practices including re-use of contaminated needles and syringes).

Several factors make it difficult to produce a vaccine against HCV, including difficulty in propagating primary isolates of viruses in vitro or to test cell infection models, a paucity of accurate animal models, difficulty in testing efficacy in humans and genetic variants of the virus keeping viral antigens as a moving target for immune responses. A number of candidate HCV vaccines have, to date, been developed, reaching various stages of progress (examples in Table 1), although none has reached licensure and/or deployment. As for all vaccines, there are several features of vaccines that need to be considered in HCV vaccine design. These include the form in which the HCV antigens are delivered (recombinant protein, nucleic acid, peptides), delivery platform (recombinant virus, liposome, lipids/detergent, virus-like particles (VLPs)), co-administered immune adjuvants (bacterial-derived molecules, aluminium salts) and vaccination regimen (prime, boost, interval, target population). Most of the proteins that HCV possesses are potential antigen candidates that could be incorporated into a vaccine (Figure 1a), namely the core (C), envelope glycoproteins E1/E2, p7 and non-structural (NS2-5) proteins, and appropriate antigens must be selected. Structural proteins such as envelope glycoproteins are targets for neutralising antibodies (NAbs), whilst conserved non-structural (NS) proteins are considered better targets for T cell responses. HCV is a hepatotropic, enveloped, single-stranded, positive-polarity RNA virus that is not directly cytolytic or cytopathic, facilitating chronicity. Currently, HCV is classified into eight genotypes (GT1-8) and more than 86 subtypes, with GT1 being the most prevalent globally. The immune response against HCV is complex and is a double-edged sword, being capable of causing immunopathology (the cascade of inflammation, fibrosis, regeneration, cirrhosis and cancer) as well as potentially providing immune protection against the virus. This is in part due to the site of viral replication, the liver, possessing features that promote immune tolerance and immune downregulation (such as via regulatory T cells), which can suppress vital antiviral immune responses. HCV is able to utilise this site of relative immune privilege to evade immunity and has evolved mechanisms by which it can circumvent immunity, including generating antigenic escape mutants. The RNA-dependent RNA polymerase of HCV lacks proofreading capacity, meaning that many mutations and, hence, many antigenic variants (quasi-species) occur.

In addition, the rational design of any HCV vaccine must utilise advances in our understanding of the unique and dynamic composition of HCV particles circulating in the blood, recently confirmed by ultrastructural studies. HCV replicates mainly in the liver and hijacks one of its major roles, lipid metabolism, both inside and outside hepatocytes. In circulation, HCV is heterogenous both in size and density and exists in at least three, possibly interchangeable particles [2] (Figure 1b, i–iii):

i. Infectious HCV lipo-viral particles (LVPs) containing HCV core encapsulating RNA, HCV envelope proteins E1 and E2 and lipoproteins, including apolipoprotein E, which is not only present in higher copy numbers than E1/E2 but also facilitates viral entry via glycosaminoglycans, Low-Density Lipoprotein receptors, CD81 and Scavenger Receptor B1. The lipids in the HCV-LVPs serve to mask neutralising antibodies (NAbs) against HCV E1/E2.
ii. Sub-viral particles which do not contain core/RNA but are associated with HCV E1/E2. They significantly outnumber HCV-LVPs and may serve as a “decoy” for NAbs.
iii. Non-enveloped core particles which, again, may divert the immune response; they have been found to be a component of cryoprecipitable immune complexes in type II mixed cryoglobulinaemia.

First-line exposure and infection with HCV-LVPs leads to hepatocyte invasion by infectious particles, where viral RNA is sensed by Pattern Recognition Receptors (PRRs); Toll-Like Receptor( TLR)3, TLR7, retinoic-acid inducible gene I (RIG-I), melanoma differentiation-associated protein 5 (MDA5), NOD-like receptors (NLRs) and RIG-I-like receptors. (RLRs), leading to expression of type I and type III interferons, interleukin (IL)-1β and IL-18. These interferons inhibit viral replication and activate Natural Killer (NK) cells capable of killing infected hepatocytes via cytolytic pathways. Local antigen-presenting cells take up released viral antigens and are activated through interferons, IL-1β and IL-18 and via PRRs, and present viral peptide epitopes to CD4^+^ T cells via Major Histocompatibility Complex (MHC) class II to generate helper responses and to CD8^+^ T cells via MHC class I to generate cytotoxic T cell (CTL) responses. Whilst HCV-specific CD8^+^ CTLs directly kill infected cells via perforin/granzyme (Figure 1c,d), HCV-specific CD4^+^ helper T cells (Th) of the Th1 phenotype secrete interferon (IFN)γ, which inhibits viral replication and activates other cells such as NK cells and macrophages, causing local inflammation. Th cells are also critical not only to the generation and maintenance of robust functional CTLs but also for the activation and maintenance of B cells that recognise and secrete NAbs. These NAbs intercept viral particles, including sub-viral particles, and may prevent infectious LVPs from infecting hepatocytes. They tend to be directed against the E2 glycoprotein HVR1 region or the AR3 and AR4 regions, making them candidates for inclusion in vaccines. Evidence for the possibility of vaccine-induced immunity comes from the observation that spontaneous viral clearance occurs in 25% of those having primary infection and in 30–60% of those having one or more reinfections. Analysis of individuals who resolved their infection demonstrates immunity that is protective, comprising broadly cross-reactive (cross-genotype) NAbs, HCV-specific CTLs and Th1 cells that are proliferative and possess memory marker CD127 and lack programmed cell death protein (PD)-1. There is a particular emphasis on memory immune responses determining the strength and longevity of immune responses, and HCV vaccines aim to emulate or exceed this. Individuals with chronic HCV demonstrate exhausted and dysfunctional T cell immunity against HCV, with HCV-specific T cells expressing PD-1, and function is typically not restored following viral cure with DAAs. Exhaustion appears to be in part related to the very high viral (and hence antigenic) loads present. It has also become apparent that the homing of T cells to the liver and the establishment of tissue-resident memory T cells (T_RM_) that possess CD69, CD186 and CD103 are important for immunity in the liver. Priming of T cells locally to the liver may imprint these cells to remain there. 

Several studies have shown that vaccines comprising the recombinant E1/E2 heterodimer together with adjuvants such as MF59 and ISCOMATRIX generated protective immunity in chimpanzees and, in humans elicited antibodies that neutralised HCV across genotypes [3]. A recent study in this journal by Ackache et al. (2019), entitled “Effect of Different Adjuvants on the Longevity and Strength of Humoral and Cellular Immune Responses to the HCV Envelope Glycoproteins” [4], compared lipid/saponin-based vaccine formulations used in licenced vaccines and a novel archaea-bacterial-derived adjuvant sulphated S-lactosylarchaeol (SLA), similar to monophosphoryl lipid A (MPL), in combination with recombinant E1/E2 heterodimer. Mice were immunised with three intramuscular inoculations (2–3 weeks apart) and immunity was successfully achieved as measured by antibody ELISA and pseudovirus inactivation (i.e., NAbs) and by T cell ELIspot and intracellular cytokine staining (for simultaneous IFNγ, TNFα and IL-2 [i.e., polyfunctionality]). Levels of immunity were maintained at 6 months post-vaccination. This is a similar immune profile to that seen in HCV-resolving humans.

Despite the initial promise of non-replicating viral-vector vaccines for HCV (Chimpanzee Adenovirus 3 (ChAd3) and Modified Vaccinia Ankara (MVA), both encoding genotype 1b NS3-5A) given in a heterologous prime–boost regime showing protective immunity in chimpanzees [5] and immunity in human trials [6], protective immunity in high-risk individuals was not achieved [7], which has stalled progress of this approach. However, the success of such an approach, particularly in the context of SARS-CoV-2/COVID-19, suggests that it could be successful for HCV if the vaccination makeup and regimen can be optimised. It may be that the antigenic component needs to better match the HCV variants in circulation and that more studies of these heterogenous HCV particles are required in fasted individuals who resolve acute infection in parallel with characterising their immune response. Other studies have used perforin as an effective adjuvant for DNA vaccines [8] or suggest the use of virus-derived exosomes as vaccine components [9]. Further approaches involve adeno-associated viral vectors, which may enhance liver-homing of T cells [10], and combination HCV/HBV vaccines that utilise the ability of HBV to form immunogenic VLPs, as shown for licenced HBV vaccines [11]. Thus, similarly for SARS-CoV-2 and another flavivirus, Zika virus, a number of effective vaccines could enter trials and become available for HCV, allowing at-risk groups or wider populations to be protected (from persistence if not from infection), which would reduce viral burden and transmission. The success of the SARS-CoV-2 RNA-based vaccines BNT162b1 (Pfizer and BioNTech) and mRNA-1273 (Moderna) with their demonstrated efficacy at preventing COVID-19 should provide new avenues to develop safe and affordable HCV vaccines adopting these exciting RNA-based approaches. Ultimately, not only must the immune system remember HCV in the correct way and scientists continue to develop HCV vaccines, but society must remember the need for a vaccine and recognise that it can be achieved if recognised as sufficiently important.

## Figures and Tables

**Figure 1:**
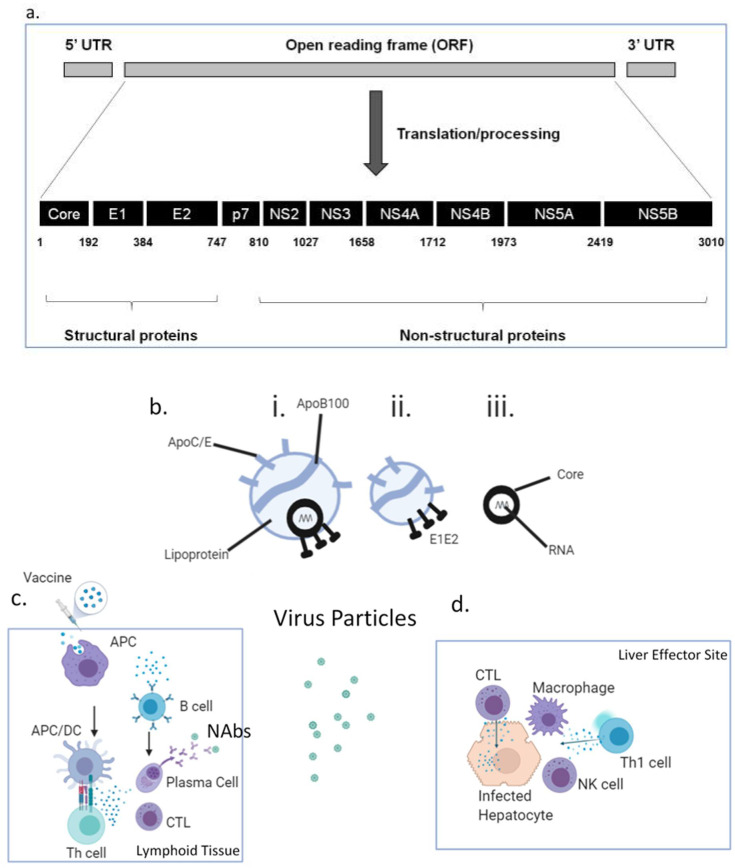
HCV proteins (antigens), dynamic composition of HCV particles, and indicative mechanisms (non-exhaustive) of vaccination and anti-viral immune protection. (**a**) The single stranded 9.6 kb RNA genome encodes a long open reading frame (ORF), which is flanked by 2 untranslated regions (5′ and 3′ UTR). The translated polyprotein is co-translationally and post-translationally processed by cellular and viral proteases. The numbers below the polyprotein denote the amino acid positions of the respective cleavage sites. (b) The unique and dynamic composition of the HCV particles circulating in the blood: (i) infectious HCV lipoviral particles (LVP), (ii) subviral particles, and (iii) non-enveloped core particles. (c) Potential vaccination process involving injection of vaccine, uptake by Antigen Presenting Cells (APCs particularly Dendritic Cells [DCs]), processing and presentation to T cells (Th cells and cytotoxic T cells (CTLs)), as well as viral recognition by B cells and the production of specific antibodies. (**d**) Potential effector mechanisms against HCV involve recognition of infected hepatocytes by CTLs and their secretion of cytolytic granules, recognition of antigen by Th1 cells and secretion of interferon (IFN)γ that activates local NK cells and macrophages. Created with BioRender.com.

**Table 1:** Examples of vaccines in development for hepatitis C virus (HCV).

Vaccine Type	Antigen	Formulation/ Vector	Phase	Immunity Generated	Protection Achieved	Industrial Developer (Previous/Current)
1. Subunit	recE1E2 heterodimer; E2; Core	MF59/ISCOMATRIX	Preclinical Phase Ib	Cross-genotype NAbs, CD4+ T cells	In Chimpanzees	Chiron/Novartis/Italy
2. Virus-like particles (VLPs)	E2; Core; HBV/HCV	VLPs	Preclinical	NAbs, CD4+T cells	In Chimpanzees	None
3. Viral vector	NS3-5	ChAd3/MVA Ad-associated virus pVax-Ns5b	Phase I/II Preclinical	CD4+, CD8+ T cells	In Chimpanzees, not in humans	Okairos/GSK/Oxford Uni/NIH/NIAID
4. Peptide	p7/p6 MAP	IC-41	Preclinical	T cells	NT	Copenhagen/SSI
5. DNA	Core; E1; E2; NS3-5A	CIGB-230 VGX-6150 Ino-8000	Preclinical Phase I Phase I	CD4+, CD8+ T cells	NT NT NT	Chiron/Novartis Cuba Inovio
6. Nanoparticles	E2	AddaVax	Preclinical	NAbs	NT	Scripps

Non-exhaustive list of vaccines in development for HCV. NT—not tested; NAbs—neutralising antibodies.

## Data Availability

No data, not applicable.
